# Supplementary material for: Molecular mechanism of quorum sensing inhibition in Streptococcus by the phage protein paratox
Source: J Biol Chem. 2021 Jul 21;297(3):100992. doi: 10.1016/j.jbc.2021.100992 (PMC8383118; doi:10.1016/j.jbc.2021.100992)
Supplement: Supplemental Figures S1–S8 and Table S1 [file mmc1.pdf]

Molecular mechanism of quorum sensing inhibition in *Streptococcus* by the phage protein paratox

Nicole R. Rutbeek[1], Hanieh Rezasoltani[2], Trushar R. Patel[3], Mazdak Khajehpour[2], and Gerd Prehna[1*]

[1]Department of Microbiology, University of Manitoba, Winnipeg, MB R3T 2N2 Canada
[2]Department of Chemistry, University of Manitoba, Winnipeg, MB R3T 2N2 Canada
[3]Department of Chemistry and Biochemistry, University of Lethbridge, AB T1K 3M4, Canada

* To whom correspondence should be addressed: G.P.
Email: gerd.prehna@umanitoba.ca
Telephone: (+1) 204-474-6543

List of Supplementary Data:

Table S1: Primers used in this study

Figure S1: Sequence conservation of Prx and ComR proteins

Figure S2: SEC binding assays of Prx with various ComR orthologs

Figure S3: SDS-PAGE gels of the SEC binding assays of the ComR minimal domains with Prx

Figure S4: Structural comparison of Prx crystal structures

Figure S5: Coomassie stained SDS-Page gels of Prx variant binding assays

Figure S6: SEC-SAXS data analysis

Figure S7: Low-resolution SAXS models of ComR, ComR:Prx, and Prx.

Figure S8: Additional structural comparison between the ComR DBD:Prx interaction and the LasR DBD:Aqs1 interaction.

Table S1. Primers used in this study

Primer Name	Sequence (5' to 3')	Source[a]
UA159 PcomS F	AAGCAGGTAGACTGCCTTCCATTGG	18
FAM-UA159 PcomS R	5'FAM-CCTGTTATTCTCCTTTCTTT	18
TPR_NdeI_F	ATTACATATGAGTCGTTATAAAGAACTAAA GTATTTATTATTACG	
TPR_BamHI_R	ATTAGGATCCTTATGTCCCGTTCT	
DBD_F	ACATTTTCTCAGTCCGTTAAATAGC	
DBD_R	TGAGTTACCCAGTCGTTATAAAG	
PrxE6A_F	TATATAGATGCGTTTAAAGAAGCGATTG	
PrxE6A_R	TAACATATGTATATCTTCTTCTTAAAG	
PrxE9A_F	GAGTTTAAAGCAGCGATTGATAAGG	
PrxE9A_R	ATCTATATATAACATATGTATATCTCC	
PrxD12A_F	ATACCCCTTAGCAATCGCTTC	
PrxD12A_R	ATTTTAGGTGACACAGTAGC	
PrxF31A_F	CGGAAAGATAGCTGATTATGTGTTACCAC	
PrxF31A_R	TTTTTACGCACTATCGCTGTG	
PrxD32A_F	AAGATATTTGCTTATGTGTTACCAC	
PrxD32A_R	TCCGTTTTTACGCACTATC	

[a]All primers are from this study unless indicated otherwise

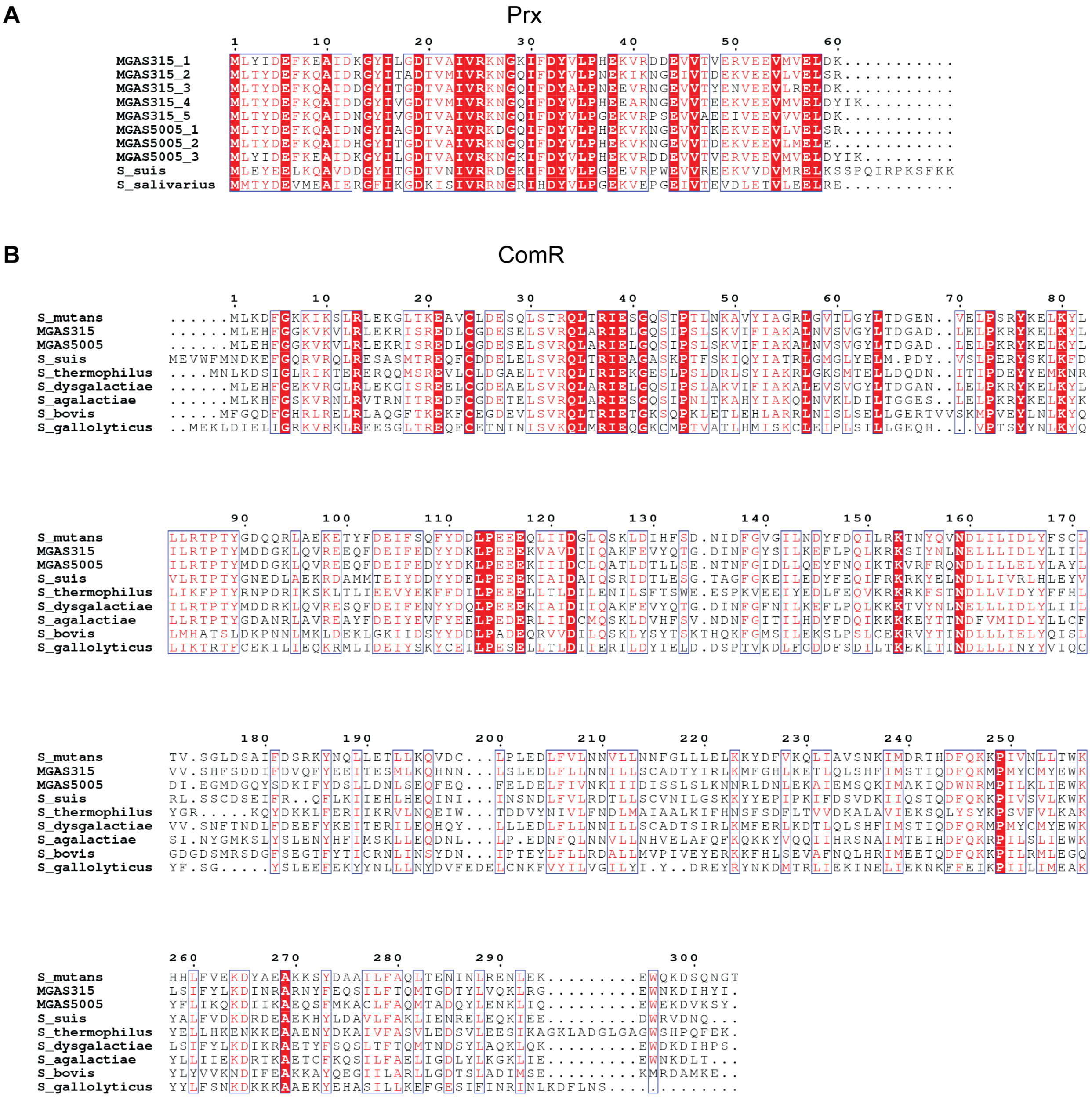

Figure S1. Sequence conservation of Prx and ComR proteins. A) Multiple sequence alignment of select Prx proteins. Each protein sequence is numbered by strain or species. For MGAS315 and MGAS5005 multiple Prx are present as indicated by number. B) Multiple sequence alignment of select ComR proteins. The DBD is approximately residues 1-66 and the TPR is approximately residues 75 to the C-terminus. Each protein sequence is numbered by strain or species. Red highlights indicate sequence identity and red letters indicate sequence homology. The alignments were made using the server clustalo (https://www.ebi.ac.uk/Tools/msa/clustalo/) and the server Espript3 (https://espript.ibcp.fr/ESPript/ESPript/).

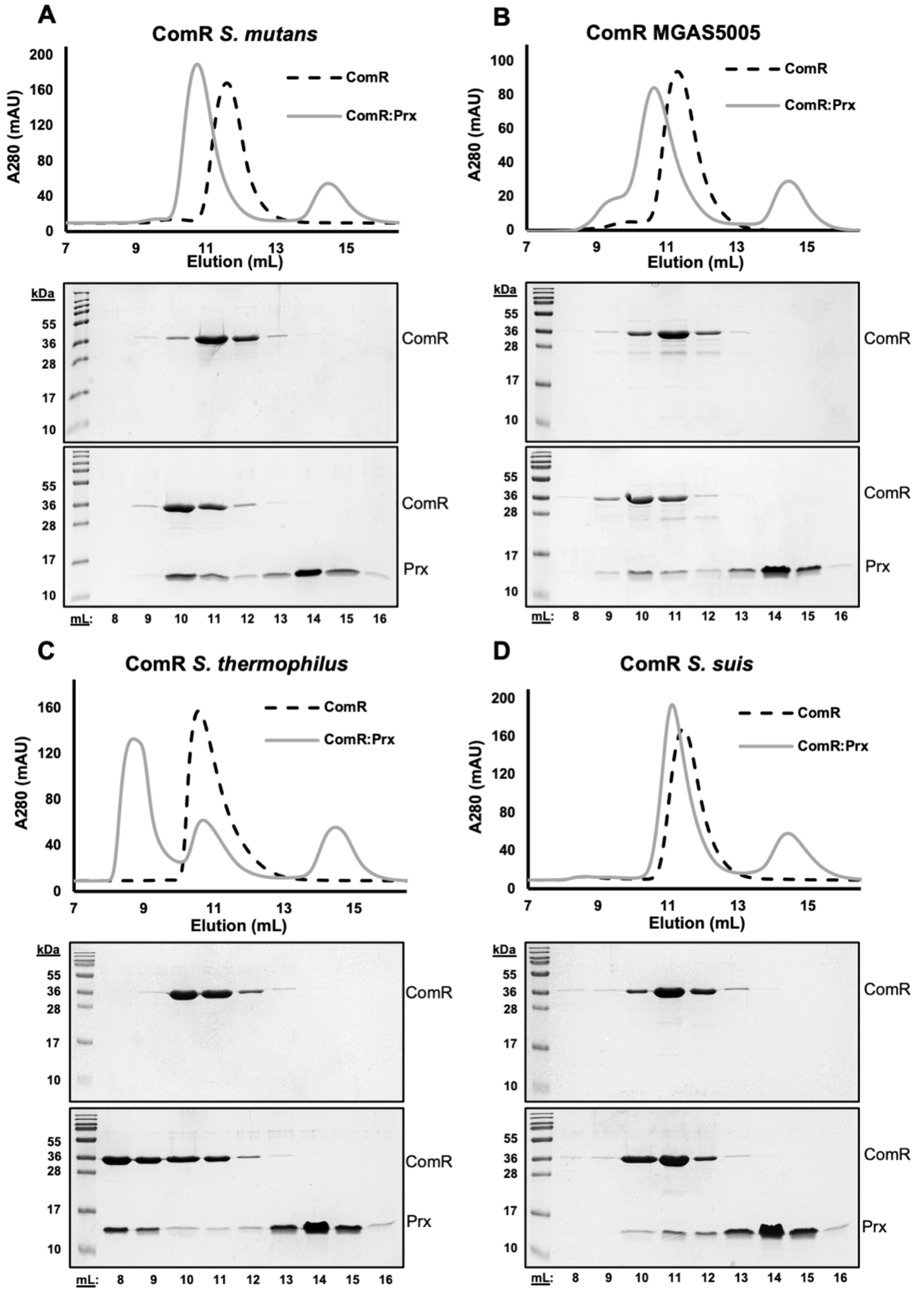

Figure S2. SEC binding assays of Prx with various ComR orthologs. A) ComR *S. mutans*:Prx, B) ComR MGAS5005:Prx, C) ComR *S. thermophilus* :Prx, and D) ComR *S. suis* :Prx. The top panel is each figure represents the SEC chromatogram and the bottom figure the Commassie stained SDS-PAGE gel of each experiment. ComR alone is represented by a dashed black line and the complex experiment a solid grey line. Fractions in mL are indicated below each gel. The void volume is approximately 8mL.

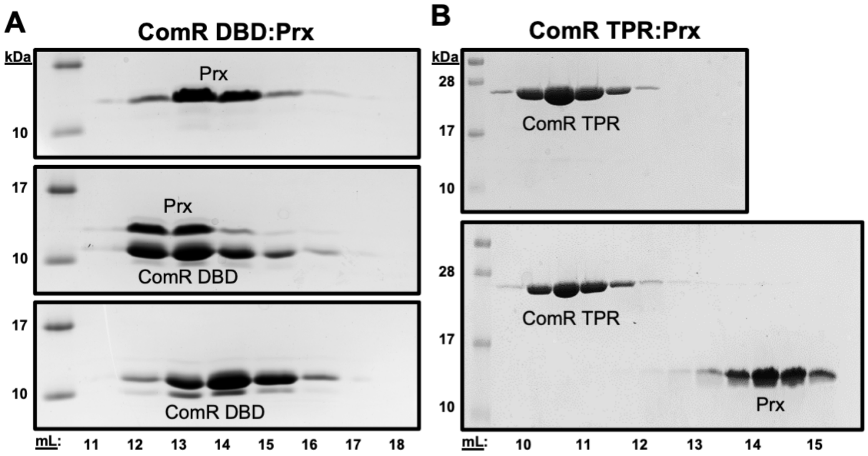

Figure S3. SDS-PAGE gels of the SEC binding assays of the ComR minimal domains with Prx. A) ComR *S. mutans* DBD and Prx. B) ComR *S. mutans* TPR domain and Prx. Corresponding fractions in mL are listed below each gel. Gels were visualized by staining with Coomassie dye.

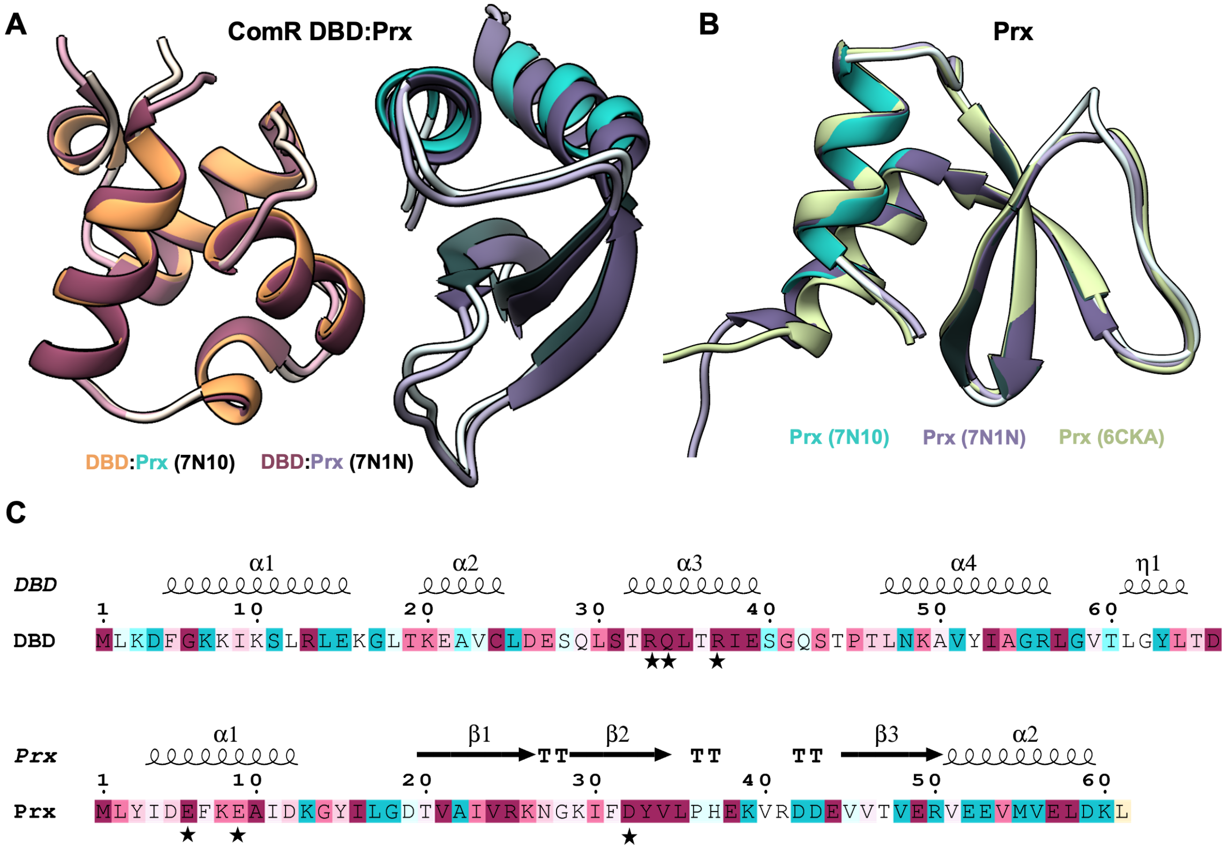

Figure S4. Structural comparison of Prx crystal structures. A). Structural alignment of two ComR DBD:Prx structures. DBD:Prx minimal domain (PDBid: 7N10) and DBD:Prx proteolytic digest (PDBid: 7N1N). B) Overlay of each Prx with the uncomplexed Prx structure (PDBid: 6CKA). C) Conserved residues of each domain plotted with secondary structure. Residues important for binding are starred. Molecular graphics were drawn using UCSF Chimera (https://www.cgl.ucsf.edu/chimera/). Sequences were plotted with Consurf (https://consurf.tau.ac.il/) and Espript (https://espript.ibcp.fr/).

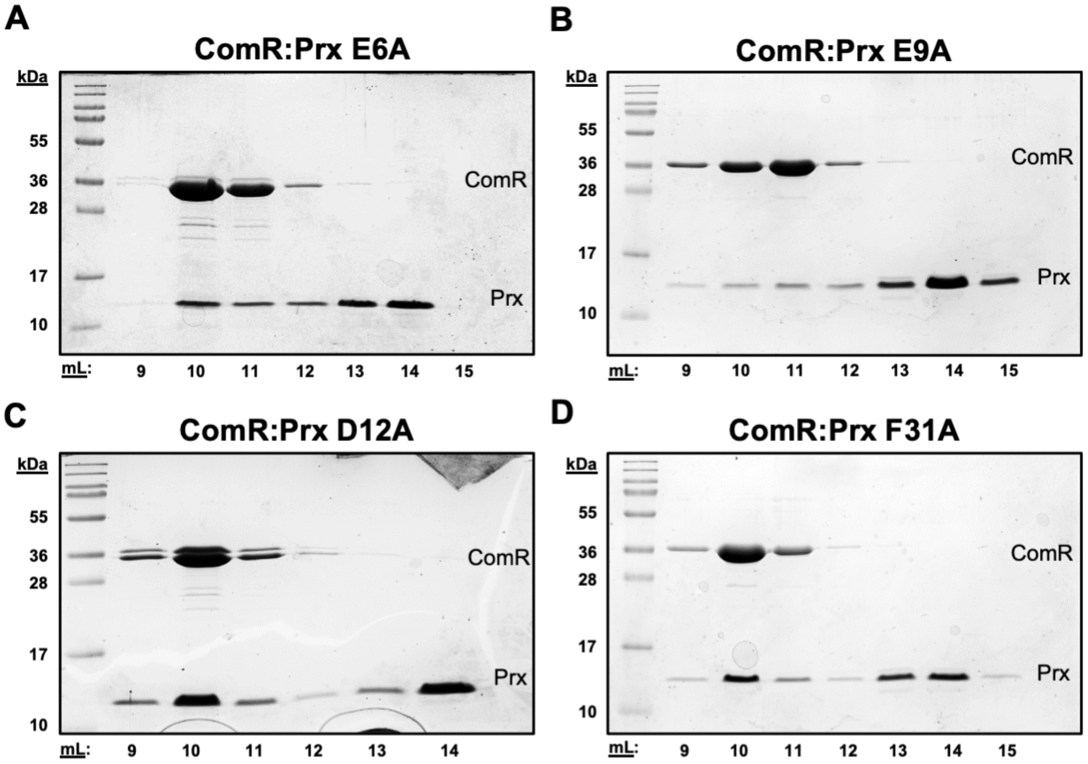

Figure S5. Coomassie stained SDS-Page gels of Prx variant binding assays. A) ComR and Prx E6A B) ComR and Prx E9A C) ComR and Prx D12A D) ComR and Prx F31A. Each well corresponds to the mL fractions of the size exclusion chromatograms in Figure 4A.

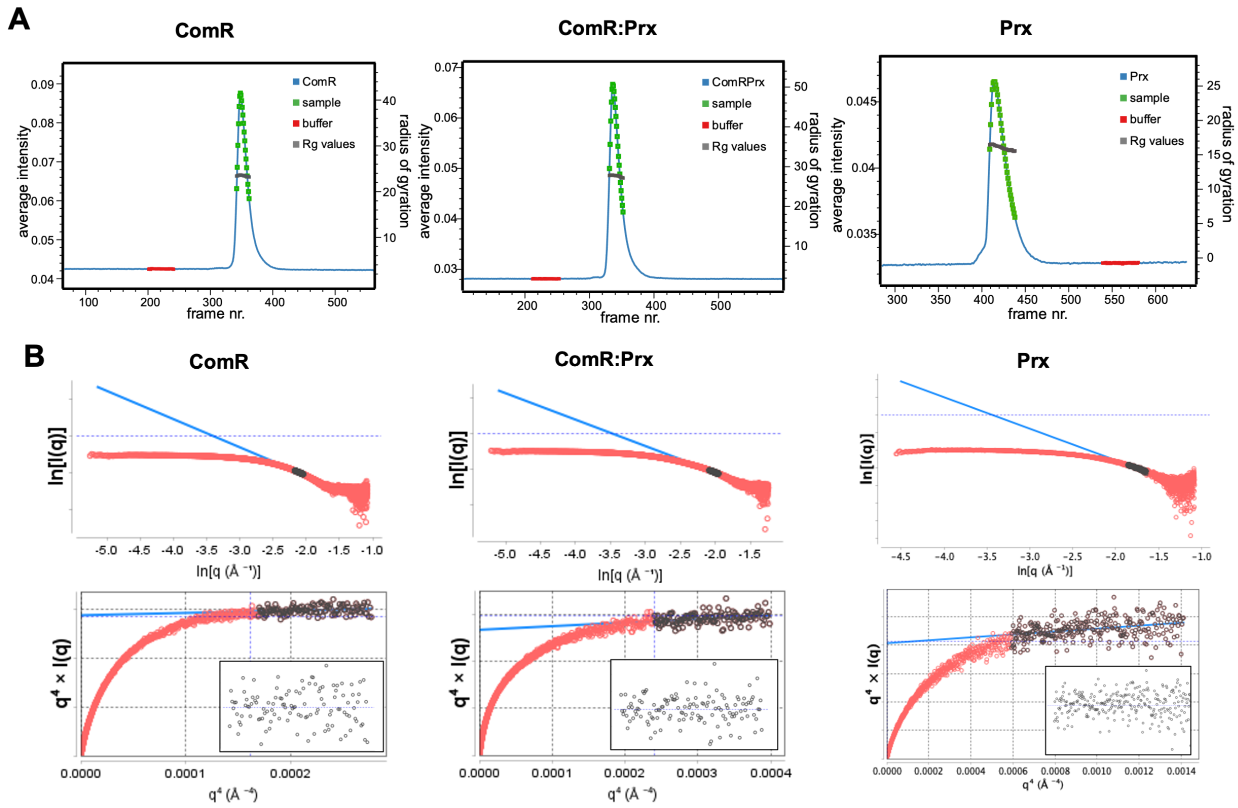

Figure S6. SEC-SAXS data analysis. A) SEC-SAXS elution profile showing the scattering intensity of each sample (ComR, ComR:Prx, Prx) along with the sample selection (green) and buffer selection (red). The Rg values are shown in gray (scale on right). B) Power law plot, along with a Porod-Debye plot, showing the linear curve fit for the determination of the Porod exponent for each protein sample. The inset of each plot highlights the even distribution of residuals.

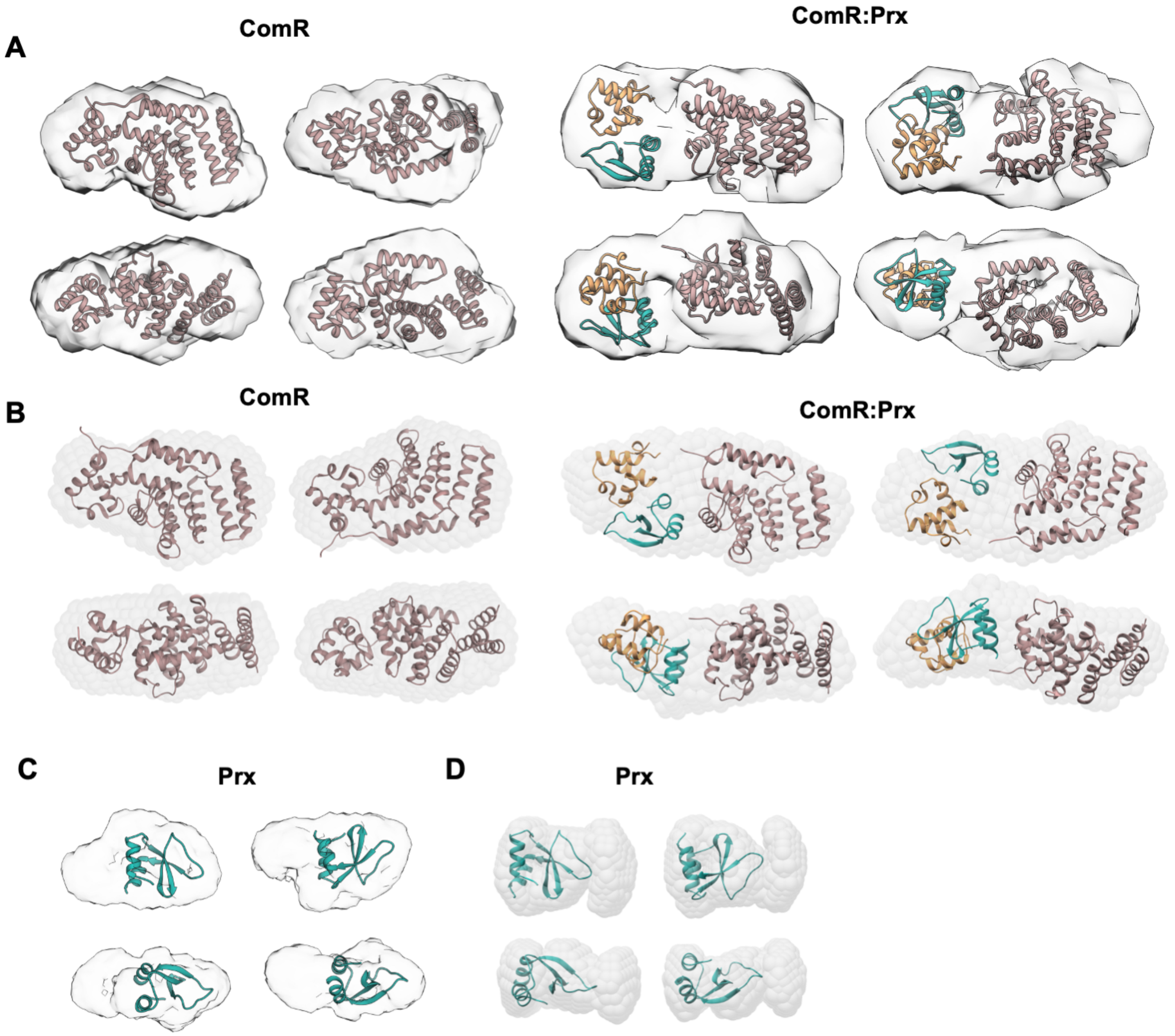

Figure S7. Low-resolution SAXS models of ComR, ComR:Prx, and Prx. A) Various images are shown of the aligned PDB structures to the low resolution electron density maps calculated using DENSS for ComR and ComR:Prx to provide a more complete representation of each fit. B) Various images are shown of the aligned PDB structures to the low resolution electron density maps calculated using DAMMIN for ComR and ComR:Prx to compare with the models provided by DENSS. C) Prx modeled into the SAXS density envelope calculated using DENSS. D) Prx placed in the SAXS enveloped calculated using DAMMIN. All modeling was performed manually using UCSF Chimera.

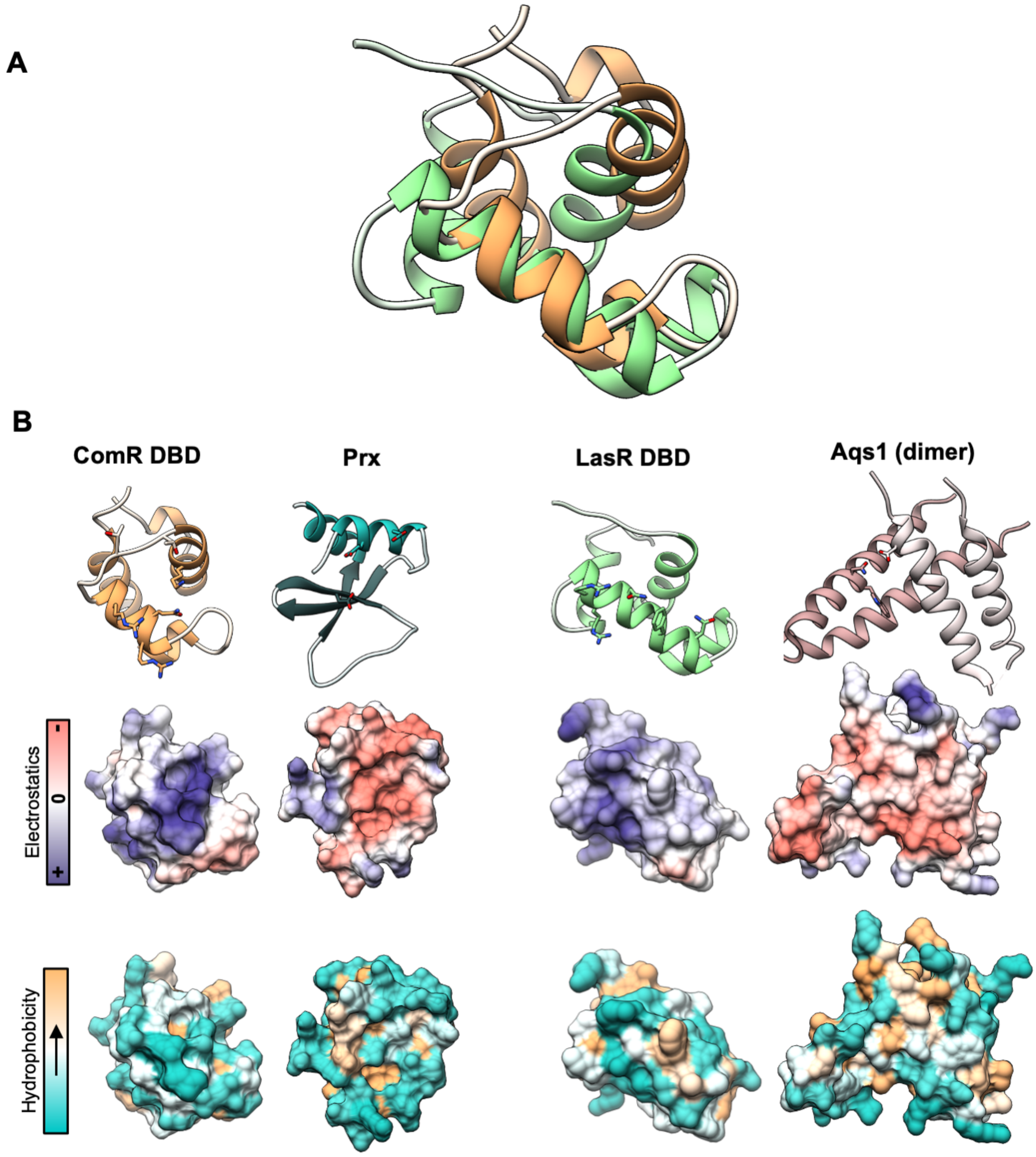

Figure S8. Additional structural comparison between the ComR DBD:Prx interaction and the LasR DBD:Aqs1 interaction. A) Structural alignment of the ComR DBD and LasR DBD. The two DNA binding domains have 16% sequence homology, an RMSD of 5.1 and Z score of 2.1. Prx and Aqs1 failed to align. B) Surface properties of both the ComR:DBD and Prx, and the LasR DBD and Aqs1. For each protein, key residues on the interaction surface are shown, along with electrostatics and hydrophobicity. Electropositive (blue), electronegative (red), hydrophobic (yellow). Molecular graphics and surfaces were drawn using Consurf (https://consurf.tau.ac.il/) and UCSF Chimera (https://www.cgl.ucsf.edu/chimera/).
